# *Acronymolpus*, a new genus of Eumolpinae, endemic to New Caledonia (Coleoptera, Chrysomelidae)

**DOI:** 10.3897/zookeys.547.9698

**Published:** 2015-12-17

**Authors:** G. Allan Samuelson

**Affiliations:** 1 J. Linsley Gressitt Center for Research in Entomology, Department of Natural Sciences, Bishop Museum, Honolulu, Hawaii 96718, U.S.A.

**Keywords:** Chrysomelidae, Eumolpinae, Eumolpini, *Acronymolpus*, new genus with 4 new species, New Caledonia

## Abstract

The genus *Acronymolpus* is proposed as new. It is represented by four new species, all of which are endemic to New Caledonia. Proposed are: *Acronymolpus joliveti* **sp. n.** (type species), *Acronymolpus gressitti* **sp. n.**, *Acronymolpus meteorus* **sp. n.**, and *Acronymolpus turbo* **sp. n.**

## Introduction

*Acronymolpus*, a new genus of Eumolpinae, is proposed herein. The four included species are new and are endemic to New Caledonia. This genus is unique among its allied Eumolpini, e.g. *Dematochroma* Baly, by having the metacoxae enlarged and nearly reaching the posterior margin of the first abdominal ventrite.

Specimens appear to be very rare in collections, with only seven individuals known to date. This study is based only on these specimens representing the four new species. The earliest examples were taken in 1963, then through later years to 2005.

## Material and methods

Collections: BPBM, Bishop Museum, Honolulu, Hawaii; USA; CXMNC, Collection Xavier Montrouzier, Institut Agronomique néo-Calédonien, La Foa, New Caledonia, with a holotype from the latter to be deposited in the MNHN, Museum of Natural History, Paris, France.

Owing to the rarity of specimens, three of the species are left intact and not compromised by dissecting.

Measurements are taken from a calibrated ocular micrometer on a Leica MZ7 stereo microscope and are reported in mm and cmm, the latter = 1/100 of a mm. Abbreviations or brief names of selected body structures are: BL body length; BB body breadth; HB head breadth; IAS transverse breadth of interantennal space; AS transverse diameter of antennal socket; ORB transverse space of orbit between antennal socket and eye; IOS shortest transverse distance between eyes; EYE maximum diameter × breadth of eye; GENA distance between genal apex and lower eye margin; PNL pronotal length; PNB pronotal breadth.

## Taxonomy Tribe Eumolpini

### Acronymolpus gen. n.

Taxon classificationAnimaliaColeopteraChrysomelidae

http://zoobank.org/80C47612-8196-4FE4-B645-8BCDA9AE49FA

#### Description.

Proepisternal margin straight; pygidial groove present; metatibial apex entire, lacking emargination; claws appendiculate.

Body fusiform, stout, with elytra strongly narrowed from robust basal region to preapex. Head: frontal surfaces flattened; postantennal swellings ± subtriangular or oblique, not conspicuous; oblique suture present, shallow internally, deeper along upper eye margin; coronal suture deep along mid-vertex; eye subovate. Antenna slender, elongate and attaining apical 1/3 or more of elytron. Pronotum convex with anterolateral area strongly descended and appearing narrowed in dorsal view; base broadly and shallowly biconvex; posterior angle small, ± dentate; lateral margin convex and narrowed anteriorly; anterior angle slightly produced, subangulate; disc moderately to strongly punctate. Scutellum small, triangular, surface nearly smooth. Elytral punctures basically arranged in regular striae but the inner discal rows quite obliterated and confused on the basal half before they become organized into straight rows apically. Elytral epipleuron narrow to preapex and continued to apex as a thin thread. Wing normally developed.

**Figure 1.**
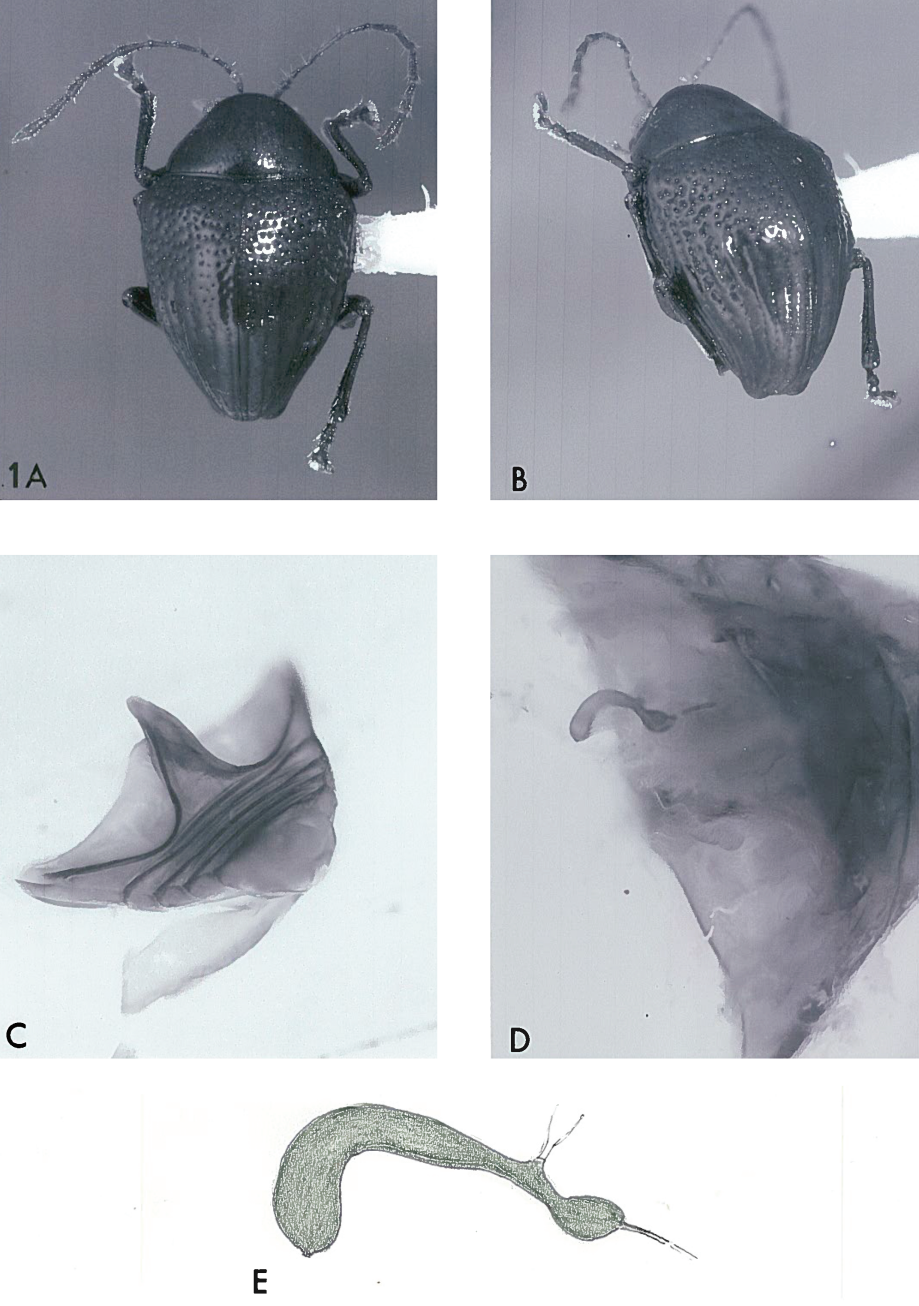
*Acronymolpus joliveti* sp. n. **A** habitus view of holotype, body length 3.3 mm **B** apical oblique view of holotype, showing apical knob-like terminations **C**.abdomen of paratype ♀, oblique ventral view showing elevated intercoxal process **D** abdomen of paratype ♀, showing position of spermatheca **E** spermatheca of paratype ♀, lateral view, length of main body 26 cmm.

Ventral surfaces: prosternum subquadrate, flattened; hypomeron ± smooth, impunctate; metasternum broadly and gently convex, ± smooth; first abdominal ventrite (Fig. 1C) not quite trisected by enlarged metacoxae on each side but on dissection with thin shelf-like extensions beneath metacoxae; intercoxal piece of forming an acute, steeply inclined triangle anteriorad; remaining ventrites strongly narrowed posteriorly, collectively subtriangular in outline. Legs: femora subclavate; metacoxa enlarged; tibiae slender, subequal to femur length.

#### Type species.

*Acronymolpus joliveti* sp. n.

#### Etymology.

*acro* (height) + *nyma* (name) + *molpus* (for *Eumolpus*); masculine.

### Acronymolpus joliveti sp. n.

Taxon classificationAnimaliaColeopteraChrysomelidae

http://zoobank.org/189F246C-8110-41AD-8878-1EDCAB384DA0

Fig. 1A–E

#### Description

(Holotype female). Body stout, fusiform, broadest across elytral humeral area, then strongly tapered to preapex. Body surfaces largely castaneous; elytron with inner interstices becoming paler orangish along apical half; antenna yellow- to orange-testaceous; legs castaneous. Dorsum glabrous; mesosternum and central part of abdominal ventrite 1 sparsely setose. Body length 3.3 mm; body breadth 2.2 mm.

Head: frontal surfaces smooth with hint of isodiametric sculpture; frons with a few large deep punctures mostly above middle; postantennal swellings ± triangular, surfaces nearly smooth; oblique suture becoming a deep sharp sulcus along upper eye margin; vertex with a few large deep punctures on each side near beginning of deep coronal suture; interantennal space flat, about 2.6 × as broad as transverse diameter of antennal socket; antennal socket and orbit with breadths subequal; interocular space about 1.4 × as broad as maximum eye diameter; eye subovate, moderately narrowed below; gena slightly over 0.6 × as deep as eye.

Antenna: slender, attaining apical 1/3 of elytron; relative lengths of segments (cmm units = 1/100 mm): 28 : 14 : 24 : 26 : 28 : 28 : 34 : 34 : 32 : 32 : 40; segments 3-6 slender, very slightly broadened apically; 7-10 distinctly heavier than preceding; last gradually thickened to apical 1/3, then narrowed to acute apex.

Prothorax: 0.57 × as long as broad; lateral margin moderately and evenly convex from base to apex; disc moderately punctate; central punctures somewhat ovate and commonly 1-2 × as large as interspaces; interspaces nearly smooth and shining with occasional micropunctures and nearly obsolete fine sculpture.

Elytron: smooth and shining; lateral margin beyond broad basal region strongly narrowed posteriorly to knob-like extremity at side of sutural apex; humerus weakly swollen, mostly smooth; discal punctures larger and deeper than pronotal ones and commonly 1-2 × as large as interspaces; interspaces commonly ± costate to subtuberculate.

Ventral surfaces: prosternum with surface dull-punctulate; hypomeron subshining, with obsolescent fine sculpture; metasternum broad, smooth-shining with fine sculpture, sparsely micropunctate; metacoxae nearly touching posterior margin of abdominal ventrite 1; relative lengths of abdominal ventrites (cmm): 48 : 10 : 10 : 12 : 20; surfaces subshining, with fine sculpture; first ventrite with median part acutely triangular and strongly inclined between coxae; last ventrite lacking median impression before apex.

Legs: slender; femora subclavate, smooth with obsolescent sculpture but sparsely punctulate; metatibia just as long as femur.

Measurements: BL 3.3 mm; BB 2.3 mm: HB 110 cmm; IAS 26 cmm; AS 8 cmm; ORB 8 cmm; IOS 58 cmm; EYE 41 × 31 cmm; GENA 26 cmm; PNB 176 cmm; PNL 102 cmm.

#### Paratype

(Female). Fig. 1D–E. Essentially identical to holotype. Spermatheca J-shaped, slender, as figured. BL 3.2 mm; BB 2.15 mm.

#### Holotype

♀. NEW CALEDONIA: Vallée d’Amoa, 7.ii.1963, C.M. Yoshimoto collector (BPBM HT16,842); Paratype ♀, Mt Panie trail, 550 m, 9.ii.1963, G. Kuschel coll. (BPBM).

#### Remarks.

Near *Acronymolpus turbo*, sp. n. in general stature, including the close proximity of the metacoxae to the apical margin of the first abdominal ventrite; both species also have ornamentation on the elytral preapex – knob-like in this species and briefly explanate in *Acronymolpus turbo*. The name honors Prof. Pierre Jolivet of Paris, who has charted our knowledge of Chrysomelidae in general and of New Caledonia in particular.

### Acronymolpus turbo sp. n.

Taxon classificationAnimaliaColeopteraChrysomelidae

http://zoobank.org/855A02E1-9776-4EAA-ABE4-46401516DF01

Fig. 2A–B

#### Description

(Holotype). Body robust, broadest across elytral posthumeral area, then strongly tapered to preapical region. Coloration reddish-piceous with paler yellowish apical elytral disc; antenna orange-testaceous. Dorsum glabrous. Body length 3.1 mm; body breadth 2.0 mm.

Head: frontal surfaces with general isodiametric sculpture; frons with several large, deep punctures above; oblique suture weak internally, becoming deeper along upper eye margin; vertex bearing several large punctures on each side of shallow coronal suture which ends near mid vertex; interantenal space rough, about 2.6 × as broad as transverse diameter of antennal socket; antennal socket and orbit subequal in breadth; interocular space about 1.3 × as broad as maximum diameter of eye; eye subovate, narrowed below; gena 0.5 × as deep as eye.

Antenna: slender, nearly reaching elytral apex; relative lengths of segments (cmm): 28 : 16 : 21 : 21 : 24 : 24 : 36 : 32 : 32 : 34 : 40; apical 2 segments heavier than preceding ones.

Prothorax: 0.55 × as long as broad; lateral margin slightly narrowed basally to mid-point, then more convexly narrowed to acutely produced anterior angle; disc closely and confusedly punctate; punctures deep and commonly 3-4 × as large as raised interspaces; only the antebasal area narrowly impunctate.

Elytron: robust basally across humeral region, then strongly narrowed posteriorly to preapical area, marked by an apical explanate margin originating at preapex of 7^th^ interstrial interval; humerus briefly pustulate and smooth, and slightly heavier than inner basal costae; disc densely punctate-subtuberculate on inner part of basal disc where punctures are confused and about 1.5 × as large as pronotal ones; elytral interstices generally swollen, with a hint of microsculpture but nearly smooth and shining.

Ventral surfaces: prosternum nearly flat, surface ± rough and punctulate; hypomeron with fairly heavy isodiametric sculpture, surface impunctate; metasternum with slightly smoother sculpture, sparsely micropunctate; metacoxa nearly touching posterior margin of abdominal ventrite 1; relative lengths of ventrites (cmm): 40 : 6 : 6 : 10 : 18; surfaces subshining with hint of sculpture and sparsely micropunctate; first ventrite strongly inclined between coxae, surface irregular slightly swollen medially on inclined part, median area apparently lacking setose patch; last ventrite lacking median impression before apex.

Legs: slender; femora weakly subclavate; metatibia linear, as long as femur.

Measurements: BL 3.1 mm; BB 2.1 mm; HB 104 cm; IAS 26 cm; AS 10 cm; ORB 10 cmm; IOS 52 cmm; EYE 40 × 34 cmm; GENA 20 cmm; PNL 92 cm; PNB 168 cmm.

**Figures 2–4.**
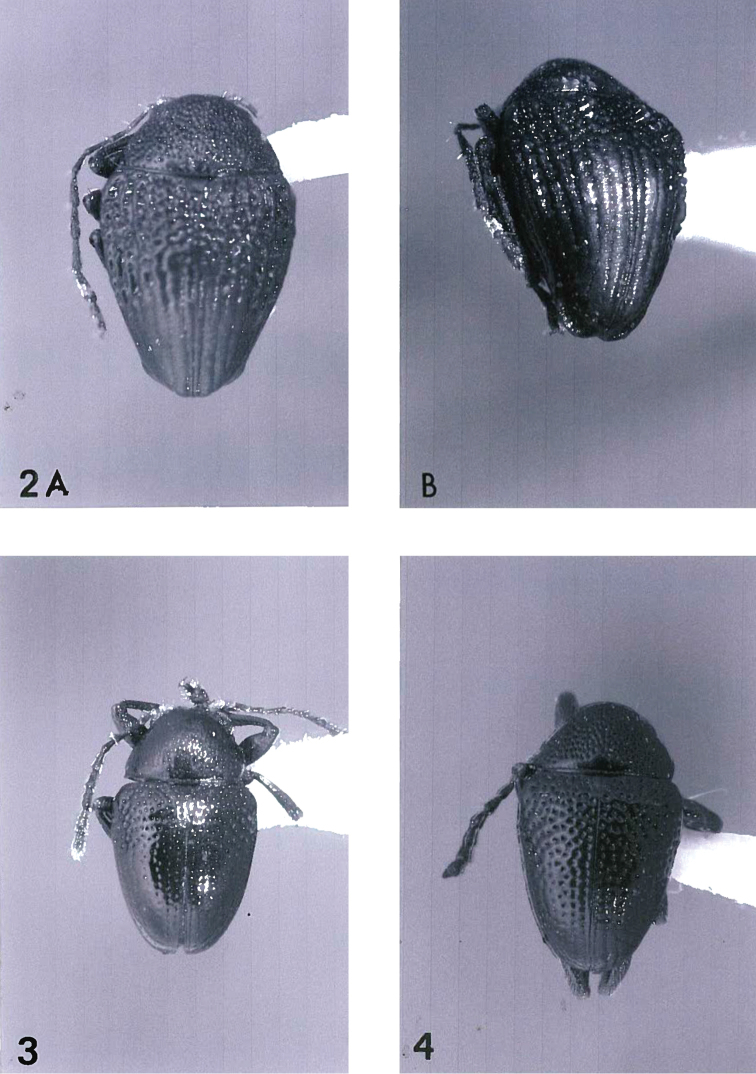
**2** *Acronymolpus turbo* sp. n. **A** habitus view of paratype, body length 3.0 mm **B** apical oblique view of paratype, showing apical explanate spoon-like terminations **3** *Acronymolpus gressitti* sp. n., habitus view of holotype, body length 2.35 mm **4** *Acronymolpus meteorus* sp. n. habitus view of holotype, body length 2.6 mm.

#### Paratype.

Essentially identical to holotype. BL 3.0 mm; BB 2.0 mm.

#### Holotype.

NEW CALEDONIA: Sarramea, Col d’Amieu, 2-23.xii.2005, Cazères, Mille, and Kataoui collectors (CXMNC/MNHN); Paratype, same locality but 2-30.xi.2005, Cazeres, Mille, and Kataoui coll. (CXMNC).

#### Remarks.

Differs further from its close relative, *Acronymolpus joliveti*, sp. n., by having closer discal puncturation of the pronotum. The name refers to the stout, ± conical form of the elytra.

### Acronymolpus gressitti sp. n.

Taxon classificationAnimaliaColeopteraChrysomelidae

http://zoobank.org/CE9C4FE9-955F-4C56-A3B6-9D0E7D70CB5F

Fig. 3

#### Description

(Holotype). Body moderately robust, broadest across elytral humeral region then moderately narrowed to rounded apex. Body surfaces and appendages largely piceous; antennal segments 1-2 yellow, 3-4 brownish; tarsal pads yellowish. Dorsum glabrous, venter: metasternum and first abdominal ventrite each with group of elongate pale setae. Body length 2.35 mm; body breadth 1.4 mm.

Head: frontal surfaces largely smooth, with a hint of fine sculpture; upper frons with a few large, deep punctures; postantennal swellings ± subquadratae; oblique suture becoming deep above eye; vertex with coronal suture deep, with surfaces on each side convexly swollen; interantennal space broad, flat, about 3 × as broad as antennal socket; orbit slightly broader than antennal socket; interocular space with breadth subequal to maximum eye diameter; gena not quite 0.5 × as deep as eye.

Antenna rather slender, attaining apical 1/3 of elytron; relative lengths of segments (cmm): 18 : 12 : 18 : 18 : 20 : 17 : 20 : 20 : 20 : 20 : 30; apical 5 segments distinctly heavier than preceding ones.

Prothorax 0.59 × as broad as long; base broadly convex across middle; lateral margin moderately convex; disc uniformly convex and rather uniformly punctured, punctures ± elliptical and commonly 1 × as large as interspaces; interspaces smooth with hint of microsculpture.

Elytron smooth and shining; lateral margin moderately narrowed from posthumeral area to preapex; apex convex: humerus slightly produced, smooth; inner basal disc with punctures deep, and larger than pronotal ones.

Ventral surfaces: hypomeron subshining, with fine isodiametrical sculpture; metastrnum with isodiametric sculpture; metacoxae enlarged and ending slightly before apical margin of ventrite 1; relative lengths of abdominal ventrites (cmm): 32 : 8 : 6 : 10 : 16; surfaces subshining, with moderate isodiametric sculpture; first abdominal ventrite moderately inclined between coxae; last ventrite with median impression before apex.

Legs: femora subclavate, surfaces with fine sculpture; metafemur and tibia subequal in length.

Measurements: BL 235 mm; BB 1.4 mm; HB 82 cmm; IAS 20 cmm; AS 6 cmm; ORB 7 cmm; IOS 36 cmm; EYE 34 × 26 cmm; GENA 16 cmm; PNL 70 cmm; PNB 118 cmm.

#### Holotype.

NEW CALEDONIA: Mt Panie, 500 m, 3.iii.1981, on *Freycinetia*, J. L. Gressitt collector (BPBM 16,843).

#### Remarks.

The less tapered body form of this species separates it from *Acronymolpus meteorus*, sp. n., which is very strongly narrowed apically. This is the only specimen of the genus with any information on plant associates; in this case *Freycinetia*. The name honors the late J. Linsley Gressitt, who contributed greatly to entomology of the Pacific and beyond.

### Acronymolpus meteorus sp. n.

Taxon classificationAnimaliaColeopteraChrysomelidae

http://zoobank.org/5B958AA0-981F-4706-821A-7A275152A777

Fig. 4

#### Description

(Holotype). Body subrobust with elytron strongly narrowed from humeral region to briefly rounded apex. Dorsal surfaces piceous; antenna with basal 3 segments orange-testaceous, remainder piceous; venter largely piceous but coxae and abdominal ventrites reddish testaceous; legs piceous. Dorsum glabrous; venter: metasternum with sparse adpressed pubescence. Body length 2.6 mm; body breadth 1.5 mm.

Head: frontal surfaces largely with fine, isodiametric sculpture; frons fairly closely punctulate above; postantennal swellings ± oblique, not conspicuous; oblique suture a fairly deep sulcus along upper eye margin; vertex below with several large punctures on each side, coronal suture deep at mid-vertex, then obsolete above; upper vertex with moderately large punctures centrally; interantennal space about 2.75 × as broad as transverse diameter of antennal socket, surface rough, punctate; antennal socket and orbit subequal in breadth; interantennal space slightly broader than maximum eye diameter (22 : 19); eye subovate; gena slightly over 0.4 × as deep as eye.

Antenna attaining apical 1/4 of elytron; relative lengths of segments (cmm): 24 : 12 : 16 : 18 : 24 : 30 : 26 : 24 : 24 : 26 : 36; apical 5 segments distinctly heavier than preceding ones.

Prothorax about 0.60 × as long as broad; lateral margin nearly straight basally before convexly narrowed anteriorly; disc closely and heavily punctate, central punctures commonly 3-4 × as broad as raised interstices.

Elytron broadest across humeral region, then subevenly narrowed to convex apex; humerus moderately produced, very briefly impunctate; basal discal punctures closely and confusedly punctate, punctures larger and rounder than the pronotal ones, and 3-4 × as large as interspaces; elytral interstices smooth-shining with hint of microsculpture.

Ventral surfaces: hypomeron impunctate but with heavy isodiametric sculpture; metasternum with finer microsculpture, obscurely punctulate; metacoxae enlarged and ending slightly before apical margin of abdominal ventrite 1; relative lengths of ventrites (cmm): 42 : 11 : 10 : 10 : 18; surfaces with moderate isodiametric sculpture; first ventrite moderately inclined between coxae; last ventrite with median impression apically.

Legs: femora nearly smooth, with fine microsculpture; metatibia as long as femur, straight, surface with duller microsculpture than femur.

Measurements: BL 2.6 mm; BB 1.4 mm; HB 90 cmm; IAS 22 cmm; AS 8 cmm; ORB 8 cmm; IOS 44 cmm; EYE 38 × 26 cmm; GENA 16 cmm; PNL 74 cmm; PNB 124 cmm.

#### Paratype.

Essentially identical to holotype; body length 2.6 mm; body breath 1.5 mm.

#### Holotype.

NEW CALEDONIA: Plateau de Dogny, 700 m, 1.ii.1963, N.L.H. Krauss collector (BPBM 16,844); Paratype, NEW CALEDONIA: Col d’Amieu, 500-600 m, 28.xii.1976, J.L Gressitt coll. (BPBM).

#### Remarks.

The uniform piceous dorsal coloration along with the closely and deeply punctate dorsal surfaces mark this species. Differs from *Acronymolpus gressitti*, sp. n. by the closer pronotal puncturation and the more narrowed elytral preapex. The name refers to the pitted surface of an iron meteorite.

### Key to species of *Acronymolpus* gen. n. and the separation of this genus from other New Caledonia Eumolpini

**Table:** 

1	First abdominal ventrite largely occupied by enlarged metacoxae; the metacoxae nearly reaching apical margin of the ventrite (Fig. 1C)	***Acronymolpus* gen. n.** 2
–	First abdominal ventrite not occupied by enlarged metacoxae; the metacoxae extending only little into basal part of the ventrite	other **Eumolpini**
2	Elytron each with apex adorned with a rounded knob-like or explanate spoon-like extension; elytral humeral area especially robust; dorsal color reddish fuscous with elytra basally darker	3
–	Elytron each normally and convexly rounded without adornments; humeral area broad but less robust; dorsal color piceous	4
3	Pronotal disc with punctures commonly 1-2 × as large as interspaces; each elytral apex with a short broad rounded tubercle (Fig. 1B); body length 3.2–3.3 mm	***joliveti* sp. n.**
–	Pronotal disc with punctures commonly 3-4 × as large as interspaces; each elytral apex with short rounded costa at side (Fig. 2B); body length 3.0–3.1 mm	***turbo* sp. n.**
4	Pronotal disc not so closely or deeply punctate; punctures commonly 1–2 × as large as interspaces; interspaces between punctures ± shining and flattened to slightly swollen; body length 2.35 mm	***gressitti* sp. n.**
–	Pronotal disc closely and deeply punctate; punctures commonly 3–4 × as large as interspaces; interspaces between punctures dull and strongly raised color; body length 2.6 mm	***meteorus* sp. n.**

## Supplementary Material

XML Treatment for Acronymolpus

XML Treatment for Acronymolpus joliveti

XML Treatment for Acronymolpus turbo

XML Treatment for Acronymolpus gressitti

XML Treatment for Acronymolpus meteorus

